# Exploring Barriers to Periodontal Treatment Adherence in Patients With Periodontitis to Inform Development of a Digital Companion: A Qualitative Study

**DOI:** 10.1111/jcpe.70044

**Published:** 2025-10-07

**Authors:** Lina Weinert, Nihad El Sayed, Michel Wensing, Bettina Dannewitz, Stefan Listl

**Affiliations:** ^1^ Heidelberg Institute of Global Health, Section for Oral Health Heidelberg University Heidelberg Germany; ^2^ Department of Periodontology, Center for Dentistry and Oral Medicine (Carolinum) Johann Wolfgang Goethe‐University Frankfurt/Main Frankfurt/Main Germany; ^3^ Department of General Practice and Health Services Research Heidelberg University Hospital Heidelberg Germany; ^4^ Medical Faculty Heidelberg University Heidelberg Germany; ^5^ Private Dental Practice Weilburg Germany

**Keywords:** behaviour change, mobile health, patient engagement, periodontal therapy, qualitative research

## Abstract

**Aim:**

The objectives of this study were to gain a comprehensive understanding of patients' barriers to adherence to periodontal treatment and to derive intervention functions for a digital companion to support them through their therapy journey.

**Materials and Methods:**

A qualitative study incorporating patient and expert interviews was designed. Data were analysed using thematic analysis. Barriers to therapy adherence and functions for a digital intervention were synthesised with the help of the Capability, Opportunity and Motivation behaviour change model.

**Results:**

Barriers related to periodontal therapy are a lack of awareness of the patients' own responsibility and prioritisation of individual oral health, lack of time to both instruct as well as learn and perform better oral hygiene and lack of knowledge about disease characteristics and ideal habits. Participants expected that a digital intervention should incorporate personalised and comprehensive educational material, possibilities to visually document progress, simple descriptions of oral hygiene tasks, reminders and the inclusion of prompts for self‐reflection.

**Conclusion:**

While a digital health companion has the potential to address the aforementioned barriers, future research should also aim to develop strategies to focus on health systems barriers (e.g., financing and workforce arrangements) as these can influence patient engagement and adherence.

## 1 Introduction

Self‐performed oral hygiene (OH) is considered crucial for successful periodontal therapy (PT) (Tonetti et al. 2015). Treatment guidelines for periodontal disease (PD) place a high priority on motivating behavioural changes for reinforcing individual OH and controlling modifiable risk factors (e.g., diet, smoking) and strengthening patients' oral health literacy (OHL). OHL refers to the capacity to obtain, process and understand information on oral health and dental care such that positive oral health behaviours are promoted (Baur et al. 2005; Firmino et al. 2018; Guo et al. 2014). Besides OHL, previous research has identified various contextual factors (e.g., limitations in quality of care or clinician‐patient communication) and individual factors (e.g., dental fear, stigma related to PD, limited locus of control, limited time, age, gender, systemic conditions, financial pressure) influencing adherence to PT (Marín‐Jaramillo and Agudelo‐Suárez 2022; Yin et al. 2022).

Behaviour change for OH is essential for successful PT, but highly complex to achieve long‐term (Tonetti et al. 2015). Oral health professionals (OHP) have a key role in motivating patients to optimise their therapeutic adherence. However, OHP cannot accompany patients daily alongside their PT journey. Hence, the role of OHP to support PD patients continuously in behaviour change is limited.

Increased patient engagement (PE) is considered a potential enabler for behaviour change and draws from four key attributes: (1) personalisation, (2) access, (3) commitment and (4) therapeutic alliance (Sanz et al. 2020; Higgins et al. 2017): Digital tools such as patient companions offer potential to operationalise such new care approaches. A systematic review (Sawesi et al. 2016) found that previous evidence is largely supportive of a positive potential of information technology on improving PE. Digital technologies, such as mobile health applications (mHealth apps), have been developed for various medical conditions (Cao et al. 2021; Väyrynen et al. 2023). Two recent systematic reviews show that most of the extant literature on dental apps focuses on caries in younger people, with several RCTs showing significant reductions in dental plaque and six RCTs demonstrating improvements in gingival health (Väyrynen et al. 2023; Toniazzo et al. 2019). Hence, while previous literature suggests some potential for mHealth to change OH behaviours, little is known about mHealth in PD and older users (Väyrynen et al. 2023). A more comprehensive understanding of patients' perceptions is paramount to align PT with patient empowerment towards optimised therapeutic adherence.

The objectives of this qualitative study were to gain a comprehensive understanding of patients' barriers to adherence to PT and to derive intervention functions for a digital companion to support them through their PT journey. The findings contribute to the optimisation of new approaches for therapeutic adherence.

## 2 Materials and Methods

### 2.1 Study Design

This study forms part of the ‘Paro‐ComPas’ project which received funding from the German Innovation Fund (Weinert et al. 2022). Drawing from detailed insights about patients' perceptions of the PT journey, the project focuses on the development and testing of an mHealth app in support of PT. Critical feedback about the potential of a digital solution was considered important for optimising the design and end‐user tailoring of the app. The project was initiated with the conservative assumption that some, but not all, patients might see potential benefits in such an app.

The qualitative study described here forms the first phase of Paro‐ComPas, which serves as a foundation for the subsequent project phases: app development, evaluation and development of an implementation roadmap (Weinert et al. 2022; also see Appendix 1 for further details).

### 2.2 Participants

Patients were recruited via six accredited academic teaching practices of the Department of Periodontology at the Center for Dental, Oral, and Maxillofacial Medicine (ZZMK), Goethe University Frankfurt am Main, Germany. Patients fulfilling the inclusion criteria (PD, age ≥ 18 years) were approached by practice personnel and were asked to fill in a sociodemographic questionnaire. Relevant experts in mHealth, dentistry and/or behaviour change were recruited via email and the professional networks of study team members (BD, SL). All the invited participants received written and verbal study information and signed an informed consent form.

### 2.3 Data Collection

Data collection took place from 07/2022 to 03/2023. Interview guides (Appendix 3) were developed by an interprofessional team (health service researchers, dentists, health economists, medical informaticians) based on a literature review and professional experience and contained questions regarding OH routines, PD knowledge, perception of care and requirements for a digital companion. Interviews were conducted via telephone or online, audiotaped and transcribed verbatim. Patient interviews were conducted by L.W.; expert interviews were conducted by B.D., N.E.S. and S.L. The end of data collection was determined upon achievement of theoretical saturation.

### 2.4 Incentives

Patients who participated in the interviews received a reimbursement of 50€.

### 2.5 Data Analysis

Thematic analysis (Braun and Clarke 2006) was chosen to code interview transcripts and to create an initial structure. Coding was performed by LW in MAXQDA 2020 (Version 20.3.0; Verbi software, Berlin, Germany) and checked by NS. Authors met regularly to discuss codes and generate ideas for interpretation. After initial coding, findings were mapped according to the COM‐B model (Michie et al. 2011), as it provides both a comprehensive way to understand behaviours and also assists in developing potentially helpful interventions, such as potential functionalities for incorporation in the digital patient companion app.

## 3 Results

Twenty‐five patients and 17 experts were successfully recruited for participation. Mean patient (expert) interview length was 28 min (39 min). Table 1 summarises patients' sociodemographic data, health behaviours, attitude towards mHealth app use and experts' expertise.

**TABLE 1:** Demographic characteristics of study participants, health behaviours and attitudes towards digital technology and mHealth app use; experts' areas of expertise.

Patients	
Adherence group	*n* (%)
Regular attendance to recall visits	17 (68)
Irregular attendance to recall visits	1 (4)
In active therapy	7 (28)
Gender	*n* (%)
Female	15 (60)
Age group (years)	*n* (%)
36–55	6 (24)
56–65	11 (44)
66–75	6 (24)
76 and older	2 (8)
Education	*n* (%)
Academic degree	10 (40)
High school education	7 (28)
Lower or intermediate secondary school	8 (30)
Self‐rated oral health	*n* (%)
Excellent	1 (4)
Very good	6 (24)
Good	9 (36)
Satisfactory	9 (36)
Affinity/interest towards digital technologies	*n* (%)
Very high	5 (20)
High	4 (16)
Mediocre	9 (36)
Low	7 (28)
Previous usage of mHealth apps	*n* (%)
No	15 (60)
Yes	10 (40)
Experts	
Area of expertise	*n* (%)
mHealth	4 (24)
Dentistry	5 (29)
Behaviour change	8 (47)
Affiliation/sector	*n* (%)
Academia	10 (59%)
Non‐university research institution	2 (12%)
Industry	1 (6%)
Professional association	3 (18%)
Private practice	1 (6%)

### 3.1 Barriers to Adherence to PT

Ten initial themes and 106 subthemes were identified. The derived code scheme was applied simultaneously to both patient and expert interviews as statements from both groups complemented each other. In a second round of coding, three main themes arose. Table 2 presents themes, subthemes and corresponding quotes.

**TABLE 2:** Themes, subthemes and corresponding quotes.

Theme/subtheme	Quote
Theme 1: Awareness regarding own responsibilities and prioritisation of individual oral health	I don't think they realize that their role is to clean along the gum line and just below the gum line, I think they think that's our job. So, for me, the thing they don't tend to understand is the cause of the condition they have, and they don't understand necessarily how important their role is. (Expert ID: 05, dentistry, academia)
Subtheme 1: Patients' active role in plaque control	[I remember an] affluent patient. Doesn't have time. And has a very grave periodontal condition and still fails to do what he needs to do. Not because he's, you know, cognitively bad or anything. But because his mindset is: “I go to the doctor; you do your part. I go in and out.” (Expert ID: 04, dentistry, academia)
	In the beginning, I didn't take it [interdental cleaning] seriously. And then, the bleeding returned. I noticed that, when I follow the routines, I just feel better. (Patient ID: 05_51, female, in supportive therapy)
Subtheme 2: Relationship with the dental team	We were able to keep all my teeth over the past 20 years (since diagnosis), just by daily oral hygiene. […] Sometimes, my dental hygienist and I tried new things, but then we reversed back if it didn't work well for us. (Patient ID: 04_67, female, in active therapy)
	You just have to understand that you must do it [interdental cleaning]. And not for four or six weeks, but for your whole life. And here it also depends on the dentist you are seeing. That they inform you, that this isn't something that will get better if you don't do anything. (Patient ID: 18_51, female, in supportive therapy)
Theme 2: Resources/capability	
Subtheme 1: Patients lack time and resources	Back in the day, I used to work a job. It was a problem back then. At your job, you can't just go and do interdental cleaning. Now, I take my time. After breakfast, I can do it. (Patient ID: 01_32, male, in supportive therapy)
	It is quite expensive. The mouth rinse. The interdental sticks aren't cheap either. And floss is costly, too. My dentist recommended me a special one I have to order online. It adds up. (Patient ID: 18_51, female, in supportive therapy)
Subtheme 2: Lack of time for instructions	So, there are reasons why communication between patient and dentist is not happening well. And one of the reasons is that normally there are no reimbursement models, so the dentist is expected to educate the patient without being compensated for their time. (Expert ID: 11, dentistry, academia)
	And they are a little bit reluctant to pay if they're a private patient or they perhaps don't feel anything has been achieved, if they come in and we talk to them about their risk factors and behaviour change and lack of control, we talk to them for half an hour. They often go away thinking “Well, I haven't had anything done, that was a waste of time or a waste of money.” (Expert ID: 05, dentistry, academia)
	They [dentists] explain it to you. You can ask questions. And as I said, also in all these preliminary consultations I had, everything was explained well and in detail. I was also informed about the risks [of the treatment] (…). So, I felt really well cared for. (Patient ID: 17_65, female, in supportive therapy)
Subtheme 3: Access to specialised dental practices	Many of them [patients] had to search for a long time to find a dentist who actually cared about this topic. They went to the same dentist for 20 years, who always said “everything is fine”. They close shop, the patient has to go somewhere else, and they say “oh, we have to extract three teeth, you have advanced periodontitis.” So, this was a common tale of woe. (Expert ID: 07, dentistry, non‐university research institution)
Theme 3: Lack in knowledge regarding periodontal disease characteristics and ideal habits	
Subtheme 1: Knowledge about disease processes and characteristics	I don't know how these bacteria got there. Of course, somebody explained it to me. It can happen through kissing or through ingestion of food. But you never get told, clearly, how you can get rid of it. Except of professional cleaning. Afterwards you know, there is a limited time in which these bacteria are eliminated, and then you start over again. (Patient ID: 05_51, female, in supportive therapy)
Subtheme 2: Understanding instructions	No, all these frills – excuse me, I wanted to say I don't use interdental brushes because I don't need them. My daughter uses this stuff, floss. My dentist also gave it to me, but I just don't need it. If I have a piece of food stuck between my teeth, I use a wooden toothpick to remove it and that's it. (Patient ID: 19_106, male, in supportive therapy)
	And media always tells you—“you have to do X” “oh no, you have to immediately stop doing X”. What used to be immensely important is considered horrible today. (Patient ID: 04_67, female, in active therapy)

#### 3.1.1 Theme 1: Awareness of Own Responsibility and Prioritisation of Individual Oral Health

All interviewed experts stressed the importance of the patients' responsibility to adhere to OH routines to sustain treatment results and to prevent disease aggravation. Yet, this high degree of individual responsibility to maintain healthy gums was often not realised by patients.

##### 3.1.1.1 Subtheme 1: Patients' Active Role in Plaque Control

Experts discussed the patients' active role in plaque control, pointing out that they only see the patient a few times a year, contrasting with patients' daily OH routines. Still, patients' awareness of responsibility for their oral health was described as low, with OHP having to do the ‘job’ of restoring and sustaining oral health. Some patients in our sample described having experienced positive changes (e.g., less bleeding; avoidance of tooth loss) by adhering to OH recommendations. Successes motivated them to stick to the newly established routines.

##### 3.1.1.2 Subtheme 2: Relationship With the Dental Team

Many patients in this sample mentioned having good ‘chemistry’ with their current dentist as a success factor in their PT. Characteristics they praised were accuracy, individualisation of treatment and time for explanations. Few patients reported dental anxiety. However, one patient recalled the dentist saying they would ‘tinker’ a new dentition, which was perceived as fear‐inducing language. Some patients used ‘we’ pronouns when discussing treatment processes, showing a strong sense of connection to the team in the dentists' practice and a sense of learning and working on OH together. Long‐term commitment to OH routines was also easier when the explanations provided by the dentist were perceived as comprehensive.

#### 3.1.2 Theme 2: Resources/Capability

To act on suggested behaviour changes, specific resources or capabilities must be available. In this sample, lacking resources related to time (both from the patients' and from the dentists' perspectives) and access to specialised practices were discussed.

##### 3.1.2.1 Subtheme 1: Patients Lack Time and Resources

For patients in this study, the newly adopted OH routine took longer than their previous routine. Fitting this routine into their schedule was sometimes described as challenging, especially by interviewees with work or childcare responsibilities. However, when starting retirement, they had more time to look after their gums. Financial restraints were also mentioned as a barrier to complying with new recommendations, for example, purchasing OH supplies or getting professional cleanings, which are out‐of‐pocket payments in Germany in most cases.

##### 3.1.2.2 Subtheme 2: Lack of Time for Instructions

Dentists in this sample reported that educating patients about PT (including education about associated conditions and implications for general health) and OH was one of their most important tasks. However, they often felt that they did not have enough time for this education or that time spent ‘only talking’ was not compensated for financially. Dentists also described that some patients did not honor the time taken for instructions either. In their experience, an appointment was only seen as valuable or billable when physical treatment was performed. Conversely, patients in this sample expressed gratitude for the thorough instructions that were provided to them and reported they had enough time to ask questions.

##### 3.1.2.3 Subtheme 3: Access to Specialised Dental Practices

Both patients and experts in this sample described changing the treating dentists towards a more specialised dentist for PT as a common occurrence. This change was often characterised by a late diagnosis of PD and a sudden need for extensive treatment. For many, the switch to a periodontist acted like a (re)start to control their disease. Yet, in some areas, these specialists were hard to find. Experts in this study also found that there are regions with profound shortages of periodontists and dental hygienists, impeding access to specialised care in Germany.

#### 3.1.3 Theme 3: Lack in Knowledge Regarding Periodontal Disease Characteristics and Ideal Habits

Lack of knowledge was commonly discussed by the experts in this sample and confirmed by patients' narratives on their OH routines and disease perceptions.

##### 3.1.3.1 Subtheme 1: Knowledge About Disease Processes and Characteristics

Patients were asked to describe PD using their own words. They commonly referred to tooth loss and symptoms such as bleeding. Some were also aware that PD is an infectious process and is considered a chronic disease. However, they also had open questions, especially regarding aetiology and secondary prevention. While few patients actively brought up links between oral and general health, experts often mentioned this aspect. They had also noticed that informing patients about these links increased patients' willingness to adequately care for their gums. The genetic component of PD was not known to many patients.

##### 3.1.3.2 Subtheme 2: Understanding Instructions

Experts noted that even after giving instructions and explanations, some patients struggled to understand the aim of interdental cleaning. Interdental cleaning was exclusively understood as a method of removing food debris and not dental plaque. Many interviewed patients also discussed confusion surrounding changing or conflicting instructions. In their experience, they had followed an OH routine for years, and suddenly new recommendations appeared in the media or were recommended by their dentists, for example, interdental brushes are better than flossing or that flossing could hurt their gums.

### 3.2 Potential Usefulness of Digital Health Companion

In general, most patients and all experts expressed interest in using or recommending a digital health companion. Table 3 presents interviewees' concrete suggestions for useful functionalities.

**TABLE 3:** Potential app functionalities as suggested by the interviewees.

Suggestion for functionality	Examples
Fun/motivational aspects	Challenges; goal setting; rewards (in‐app rewards as well as financial bonuses in real life); small games (e.g., quizzes)
Support for appointments	Reminders for existing appointments; possibility to make new appointments through the app; summary of past and upcoming appointments (e.g., what will happen in the next appointment, what to look out for after the appointment)
Support for daily oral hygiene	Brushing timer; diary (e.g., possibility to document and look back on oral hygiene routines and a place to note down symptoms); oral hygiene reminders
Tailoring/Individualisation of app content	Visualisation of individual progress; presentation of treatment successes; individual clinical measures in the app
Photo documentation	Possibility to save a photo in the diary
Direct interaction with the treating dentist	Teledentistry; possibility to chat online with practice team/dentist
Disease information	Use of attractive and varied ways to present information (e.g., short information snippets, clarifying myths about periodontal disease)

### 3.3 Synthesis According to the COM‐B Model for Behaviour Change

**FIGURE 1:**
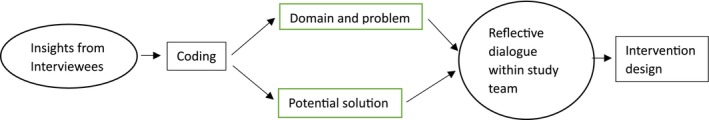
Visualisation of development progress for potential solutions and intervention functions.

Individual codes were assigned to the ‘domain and problem’ or ‘potential solutions’ categories of the COM‐B model (Michie et al. 2011) and its three domains (capability, opportunity, motivation), based on proposals by the interviewees and reflective dialogue with the study team. Figure 1 visualises this process. Table 4 presents these domains as well as potential intervention functions.

**TABLE 4:** Barriers to following oral hygiene recommendations, possible solutions and intervention functions.

Domain and problem	Potential solutions to overcome barriers	Intervention function
Capability		
Knowledge about disease processes	Information about disease characteristics	Education
Understanding instructions	Interact with information and instructions in a meaningful way	Training
Changing and seemingly contradictory recommendations	Provision of unambiguous information	Education
Opportunity		
Patients lack time due to other responsibilities	Simplification of tasks; higher priority in daily routine	Persuasion
Cost of buying tools and professional cleaning	Reimbursement through statutory health insurance	Environmental restructuring
Dentists lack time for instructions	Reimbursement through statutory health insurance	Incentivisation Environmental restructuring
Patients do not understand the importance of instructions	Education about the importance of accurate instruction	Persuasion
Difficulty in finding information about specialised practices	Transparent information about the locations of specialised practices	Enablement
Motivation		
Patients' realisation of their active role	Patient as the protagonist in their treatment through oral hygiene at home	Training
Lack of awareness of general health implications of periodontitis	Emphasising positive effects of improved oral hygiene on general health	Education

## 4 Discussion

Drawing from qualitative insights, the findings of this study suggest that one of the most important barriers to adherence to PT is a lack of awareness of patients' own responsibility and prioritisation of individual oral health. Patients often overlooked their active role in plaque control and saw dentists as the main character in their OH, neglecting the importance of daily habits. Simultaneously, experiences of success motivated patients to stick to new routines and prioritise these routines in daily life. Another barrier was a lack of time to both instruct as well as learn and perform better OH, especially because dentists and hygienists were not reimbursed for instructions. Furthermore, a lack of knowledge regarding PD characteristics and ideal habits was discussed. Patients questioned aetiology and prevention, whereas experts observed little knowledge about the connections between periodontal and general health.

In regard to the COM‐B model (Michie et al. 2011), the findings of the present study add to the extant literature as follows:

*Capability*: The three main barriers in this category (knowledge about PD, understanding instructions, interacting with changing and seemingly contradictory recommendations) can be interpreted as aspects of OHL. Previous interventions to improve OHL comprise motivational interviewing, oral health educational programmes based on cognitive behavioural therapies and the use of self‐inspections/videotapes (Carra et al. 2020). These interventions yielded positive impacts on patients' behaviours, sense of self‐efficacy and a reported higher frequency of OH. Some caution should, however, be applied when interpreting these findings, as pooled data analysis did not seem to show statistically significant differences between intervention and control groups (Carra et al. 2020). Notably, they were delivered in health care settings and no intervention made use of mHealth. MHealth has the potential to combine benefits of previous interventions, enabling use at home at self‐determined times. To harvest maximum benefits in line with the personalisation dimension of the PE concept, we recommend the inclusion of individually tailored educational material as well as progress visualisations as intervention functions in digital companions (Higgins et al. 2017).

*Opportunity*: In line with previous literature (Tseng et al. 2021), expert interviewees explained that dentists lack time for comprehensive OH instruction, a problem exacerbated by the perception that they were not financially compensated for giving instructions. The cost of supplies and professional cleanings was perceived as a barrier by patients and experts, which also concurs with previous literature (Hoben et al. 2017; Yin et al. 2022). Hence, despite generally ample dental coverage in Germany (Ziller et al. 2015), financial barriers seem to persist for PT. Besides financial barriers and aligning with previous literature (Sbaraini et al. 2012), participants also mentioned that patients may face conflicting personal priorities which can lead to low prioritisation of OH. To this end, digital companions with nudging functionalities (e.g., push notifications) might help simplify complexities for patients and stimulate positive behaviour change.

*Motivation*: Expert participants recalled that patients often would not realise their role as the protagonist in their PT journey and would not always be aware of detrimental impacts of PD on general health. Some patients recalled experiencing positive changes when adhering to routines, motivating them to continue. This realisation of self‐effectiveness was also reported in earlier studies (Sbaraini et al. 2012). To encourage patients' reflection on their routines and promote self‐effectiveness, we propose the inclusion of prompts for self‐reflection in digital companions, such as asking users to document their mouthfeel. In addition, we suggest that digital companions include compelling educational material on the detrimental impacts of PD on general health.

### 4.1 Limitations

The present study is not without limitations. Patients were recruited from specialised academic teaching practices in Germany. Future studies should aim to recruit participants representing a broader spectrum of patient perspectives. Furthermore, despite efforts taken to minimise selection bias, it is possible that patients who have fewer difficulties with the adoption of new OH routines were more likely to participate in this study. Person non‐response could partly be due to stigma surrounding impaired oral health (Doughty et al. 2023).

Nevertheless, the findings of this study provide unique and novel insights for optimising interventions to enhance adherence to PT. Particular strengths include: first, the patient perspective was identified as the guiding principle and basis for developing a novel digital intervention. This motivated the adoption of an innovative patient‐centred approach for mHealth intervention design. Second, a comprehensive behaviour change model was incorporated to complement participant perspectives with theory. Third, the study participants were mostly older persons aged 66+ which represents the patient group affected the most by PD (Eke et al. 2016). This age group, while experiencing specific challenges in handling digital tools (Baig et al. 2019), is understudied and less likely to be included in studies on digital tools (Kokorelias et al. 2022; Krukowski et al. 2024), making the chosen sample an asset of this study. Moreover, the multidisciplinary composition of the study team should also be mentioned as a strength.

The present study further endorses the high relevance of comprehensive PE in the development and evaluation of digital solutions. In the present study, patient involvement was limited to qualitative interviews with a relatively broad focus prior to software development. Future research is encouraged to engage patients continuously throughout all steps of needs assessment, software development and evaluation.

## 5 Conclusion

Through interviews with patients and experts and supported by a behaviour change model, this study identified barriers to adherence to OH recommendations which enabled identification of intervention functions for a digital companion. The authors recommend incorporating personalised and comprehensive educational material, visual progress documentation, simple descriptions of OH tasks, nudges in the form of push notifications and the inclusion of prompts for self‐reflection. However, not all identified barriers can be addressed through a digital health companion. Health systems barriers, such as lack of time and financial barriers in access to PT, persist. Future research should also aim to develop strategies to focus on health system aspects, as these can influence PE and adherence.

## Author Contributions

Lina Weinert contributed to conception, design, data acquisition, analysis and interpretation, drafted and critically revised the manuscript. Nihad El Sayed contributed to design, data acquisition, data analysis and critically revised the manuscript. Michel Wensing critically revised the manuscript. Bettina Dannewitz contributed to conception, design, data acquisition and critically revised the manuscript. Stefan Listl contributed to conception, design, data acquisition and interpretation, critically revised the manuscript. All authors gave final approval and agreed to be accountable for all aspects of the work.

## Ethics Statement

This study was conducted according to the Declaration of Helsinki, and the design was approved by the Ethics Committees of the Medical Faculty of the University of Heidelberg (S‐398/2022) and the Goethe‐University Frankfurt (2022–799), Germany.

## Consent

Before participation, both patients and experts received written and verbal information about the study and were asked to sign an informed consent form.

## Conflicts of Interest

The authors declare no conflicts of interest.

## Supporting information


**Data S1:** jcpe70044‐sup‐0001‐Appendix4.pdf.

## Data Availability

The data that support the findings of this study are available on request from the corresponding author. The data are not publicly available due to privacy or ethical restrictions.

## Appendix 1 Detailed Materials and Methods

### Study Design

This qualitative study forms the first phase of the Paro‐ComPas project, which aims to caption the diversity in patients' perspectives on their journey through PT within the context of the German statutory healthcare system and to decipher the requirements for a digital health companion for periodontitis. Paro‐ComPas encompasses three further phases: (1) development of a digital companion, (2) RCT in seven university dental clinics in Germany and (3) exploration of possibilities to transfer the digital intervention to routine care (Weinert et al. 2022).

The study is reported following the Standards for Reporting Qualitative Research Guideline (Brien et al. 2014) (Appendix 2). This is a detailed version of the material and methods. For a short version, see original manuscript.

### Participants

Patients were recruited in six accredited academic teaching practices of the Department of Periodontology at the Center for Dental, Oral, and Maxillofacial Medicine (ZZMK), Goethe University Frankfurt am Main, in Germany. Patients from the existing patient base fulfilling the inclusion criteria (diagnosis of periodontal disease, age > 18 years, ability to give informed consent) were approached by practice personnel and asked to fill in a sociodemographic questionnaire. Potential interview participants were identified following a maximum variation purposive sampling strategy (Suri 2011) aiming for both sociodemographic diversity and variation in treatment phases (supportive or active therapy).

Potential expert interviewees with relevant expertise were identified using purposive sampling through professional networks of study team members and approached via email (BD, SL). Three main areas of expertise were identified as especially relevant for the research question (mHealth, dentistry, behaviour change). Regarding institutional or professional affiliation, we aimed to recruit national and international experts from academia, research institutes, industry and health policymaking.

Before participation, both patients and experts received written and verbal information about the study and were asked to sign an informed consent form.

### Data Collection

Data collection took place from 07/2022–03/2023. Interview guides (Additional file 3) contained questions regarding OH routines, knowledge about periodontitis, perception of care and requirements for a digital companion. The interview guides were developed by an interprofessional team (health service researchers, dentists, periodontitis, health economists, medical informaticians) based on a review of related literature and professional experience. The interview guide underwent cognitive pretesting (Lenzner et al. 2016) with two laypersons. Patient interviews were conducted via telephone (outside of the therapy/practice setting), audiotaped and transcribed verbatim. Patient interviews were conducted by LW, who is a health services researcher with experience in qualitative interviewing. Expert interviews were held online, facilitated by LW and conducted by BD, NS and SL, who have expertise as dentists/periodontists and in oral health systems. All interviews were conducted as individual interviews with only one interviewee. The end of data collection was determined upon achievement of theoretical saturation as identified by consensus by the study team members.

### Incentives

Patients who participated in the interviews received a reimbursement of 50€.

### Data Analysis

Thematic analysis (Braun and Clarke 2006) was chosen to code interview transcripts and to create an initial structure, allowing for both inductive and deductive coding. Coding was performed by LW in MAXQDA 2020 (Version 20.3.0; Verbi software, Berlin, Germany) and checked by NS. Authors met regularly to discuss codes and generate ideas for interpretation.

After initial coding, individual codes were assigned to the ‘domain and problem’ or ‘potential solutions’ categories of the COM‐B model (Michie et al. 2011). The COM‐B model was chosen as it provides both a comprehensive way to understand behaviours but also assists in developing potentially helpful interventions, such as potential functionalities for incorporation in the digital patient companion app.

### Trustworthiness

In the design of this study, the four components of trustworthy qualitative research (Guba and Lincoln 1981; Shenton 2004) were incorporated. To achieve credibility, debriefing sessions within the study team were held, and an appropriate analysis method was used. The interviewer specifically introduced themselves as not being a dentist and not being in contact with a patients' treating dentist, to ensure honesty and open dialogue (Shenton 2004). Transferability was achieved by recruiting patients from practices with different characteristics (urban/rural; small/large practices) and by including (inter‐)national experts. The dependability criterion was addressed by the detailed description of the used method and by having two groups of interviewees, allowing for overlapping of results (Shenton 2004). Confirmability is ensured by an audit trail created using specialised software for qualitative research.

## Appendix 2 Standards for Reporting Qualitative Research (SRQR)

**Table:**

No.	Topic	Item	Reported on page no.
Title and abstract			
S1	Title	Concise description of the nature and topic of the study identifying the study as qualitative or indicating the approach (e.g., ethnography, grounded theory) or data collection methods (e.g., interview, focus group) is recommended	1
S2	Abstract	Summary of key elements of the study using the abstract format of the intended publication; typically includes objective, methods, results and conclusions	3
Introduction			
S3	Problem formulation	Description and significance of the problem/phenomenon studied; review of relevant theory and empirical work; problem statement	4
S4	Purpose or research question	Purpose of the study and specific objectives or questions	4
Methods			
S5	Qualitative approach and research paradigm	Qualitative approach (e.g., ethnography, grounded theory, case study, phenomenology, narrative research) and guiding theory if appropriate; identifying the research paradigm (e.g., positivist, constructivist/interpretivist) is also recommended	4–5; Appendix 1
S6	Researcher characteristics and reflexivity	Researchers' characteristics that may influence the research, including personal attributes, qualifications/experience, relationship with participants, assumptions, or presuppositions; potential or actual interaction between researchers' characteristics and the research questions, approach, methods, results, or transferability	Appendix 1
S7	Context	Setting/site and salient contextual factors; rationale^a^	5; Appendix 1
S8	Sampling strategy	How and why research participants, documents, or events were selected; criteria for deciding when no further sampling was necessary (e.g., sampling saturation); rationale^a^	5; Appendix 1
S9	Ethical issues pertaining to human subjects	Documentation of approval by an appropriate ethics review board and participant consent, or explanation for lack thereof; other confidentiality and data security issues	2
S10	Data collection methods	Types of data collected; details of data collection procedures including (as appropriate) start and stop dates of data collection and analysis, iterative process, triangulation of sources/methods and modification of procedures in response to evolving study findings; rationale^a^	5; Appendix 1
S11	Data collection instruments and technologies	Description of instruments (e.g., interview guides, questionnaires) and devices (e.g., audio recorders) used for data collection; if/how the instrument(s) changed over the course of the study	5; Appendix 1 and 3
S12	Units of study	Number and relevant characteristics of participants, documents, or events included in the study; level of participation (could be reported in results)	5; Appendix 1
S13	Data processing	Methods for processing data prior to and during analysis, including transcription, data entry, data management and security, verification of data integrity, data coding and anonymisation/deidentification of excerpts	5; Appendix 1
S14	Data analysis	Process by which inferences, themes, etc., were identified and developed, including researchers involved in data analysis; usually references a specific paradigm or approach; rationale^a^	5; Appendix 1
S15	Techniques to enhance trustworthiness	Techniques to enhance trustworthiness and credibility of data analysis (e.g., member checking, audit trail, triangulation); rationale^a^	Appendix 1
Results/findings			
S16	Synthesis and interpretation	Main findings (e.g., interpretations, inferences and themes); might include development of a theory or model, or integration with prior research or theory	5–9, Tables 3 and 4
S17	Links to empirical data	Evidence (e.g., quotes, field notes, text excerpts, photographs) to substantiate analytic findings	Table 2, Supporting Information
Discussion			
S18	Integration with prior work, implications, transferability and contribution(s) to the field	Short summary of main findings; explanation of how findings and conclusions connect to, support, elaborate on, or challenge conclusions of earlier scholarship; discussion of scope of application/generalisability; identification of unique contribution(s) to scholarship in a discipline or field	7–9
S19	Limitations	Trustworthiness and limitations of findings	8–9
Other			
S20	Conflicts of interest	Potential sources of influence or perceived influence on study conduct and conclusions; how these were managed	2
S21	Funding	Sources of funding and other support; role of funders in data collection, interpretation and reporting	1

^a^ The rationale should briefly discuss the justification for choosing that theory, approach, method, or technique rather than other options available; the assumptions and limitations implicit in those choices; and how those choices influence study conclusions and transferability. As appropriate, the rationale for several items might be discussed together.

## Appendix 3 Interview Guides

### Patients

**Table:**

Introduction
Thank you very much for taking the time for the interview today! The interview will be recorded electronically and then transcribed in pseudonymised form. Once the transcription is complete, the audio recording will be deleted. Information about places or people that you provide in the interview will be anonymised. This means that your statements can no longer be traced back to you personally. The findings or your statements from the interview will be treated confidentially and will not be shared with your dentist. There are no ‘right’ or ‘wrong’ opinions in interviews. We are interested in your personal opinions and experiences. Occasionally we will ask follow‐up questions. Do you have any questions about the process before I start the recording? How did you hear about the study? What did you find interesting about it? Why are you taking part?
2 Knowledge about the disease
What do you understand by the term ‘periodontitis’? To what extent might there be aspects of your gum condition that are unclear to you?
3 Oral hygiene routines
Please describe what oral hygiene routines you have. (e.g., how often do you brush your teeth etc., do you use aids such as interdental brushes, dental floss…) To what extent are you satisfied with these routines? To what extent are there any areas where things are not going so well? Why? What is the biggest barrier to implementing the recommendations?
4 Perception of care: accessing care and barriers
Please describe how you perceive the treatment of your gum condition. What is going well? What is going less well? Where might there be room for improvement?
5 Wishes, needs and requirements for support through a digital health companion
It can be difficult to follow all the recommendations for better oral health. Where do you see a need for support? If you see a need: What do you think of the idea of getting this support through a digital companion, for example, an app? If there is no need: What might other patients need support with? To what extent might there be other areas outside of oral hygiene, for example, organisation/appointments, information, where you need support? What do you think of the idea of receiving this support via a digital companion, for example, an app? What wishes would you have for such an app? How should this app be designed so that you enjoy using it? In addition to [recommendations for oral health/organisation of treatment/…adapt depending on what was suggested], what role does the possibility of networking with other affected people play for you?
6 Closing
What are further recommendations on this topic? Do you have thoughts you would like to share on this topic?
Thank you for this insightful conversation and your contributions!

### Experts

**Table:**

Introduction
Thank you very much for taking the time for the interview today! The interview will be recorded electronically and then transcribed in pseudonymised form. Once the transcription is complete, the audio recording will be deleted. Information about places or people that you provide in the interview will be anonymised. This means that your statements can no longer be traced back to you personally. There are no ‘right’ or ‘wrong’ opinions in interviews. We are interested in your personal opinions and experiences. Occasionally we will ask follow‐up questions. Do you have any questions about the process before I start the recording? Another note on the structure of the interview: in the first part, we would like to approach the topic with you and take a rather broad view. The second part will then deal with concrete ideas. Do you have any questions about the process? Opening question Dentists: In your opinion, to what extent is periodontitis sufficiently perceived by the public? B mHealth experts: Please briefly describe your experience with digital applications in the field of health. C Psychology: Please briefly describe your experience in the area of behaviour change. Optional: How do approaches to behaviour change differ at the individual vs. population level?
2 Effectiveness of periodontal therapy/digital support options
How do you rate the effectiveness of current care? From your point of view ‐ what are the reasons for successes and failures in periodontitis therapy? What role do the general conditions of the health care system (e.g., remuneration) play? And what role do patient‐specific factors (e.g., adherence) play? How do you rate the use of digital support by patients? What reasons do you see for a low/high take‐up? How can patients be supported in changing their behaviour, for example, implementing recommendations for better oral hygiene? In your opinion, to what extent can digital tools support, for example, apps, contribute to behavioural change in patients?
3 Perception of patients
In general, what influences patients' adherence to oral hygiene recommendations? How could adherence be improved? On which aspects of periodontal therapy do patients have a particularly high need for information/do questions arise frequently? How do patients differ as app users from ‘normal’ users? What generally influences the adherence of patients to health recommendations? How could adherence be improved?
4 Possible measures at the level of care to improve and adapt to patients' needs
How could the care of patients with periodontitis be improved? To what extent could digital support options, for example, apps, improve care? Which functions would be particularly helpful? In your experience, how should an app be designed for patients with periodontitis? What features have been shown to be helpful in similar patient populations? Optional: Point out the special features of Paro.‐Pat., for example, long freedom from symptoms. To what extent can apps support behaviour change? What special requirements might there be for a condition like periodontitis? Optional: Point out again the special features of Paro. ‐Pat., for example, long freedom from symptoms.
5 Closing
What other recommendations do you have on this topic? What other thoughts would you like to share?
Thank you very much for the open conversation and your interesting insights!
