# Supplementary material for: Exploring Barriers to Periodontal Treatment Adherence in Patients With Periodontitis to Inform Development of a Digital Companion: A Qualitative Study
Source: J Clin Periodontol. 2025 Oct 7;53(1):58–71. doi: 10.1111/jcpe.70044 (PMC12695452; doi:10.1111/jcpe.70044)
Supplement: Supplementary file 1 — Data S1: jcpe70044‐sup‐0001‐Appendix4.pdf. [file JCPE-53-58-s001.pdf]

Weinert L, El Sayed N, Wensing M, Dannewitz B, Listl S. Exploring barriers to periodontal treatment adherence in patients with periodontitis to inform development of a digital companion: A Qualitative Study: Appendix 4

Coding tree

**Codes and subcodes**	**Explanation**	**Anchor quote**
**Periodontitis pathology**	Codes relating to periodontitis as a disease. Participants' perspective and more general remarks on the disease.	
Symptoms	Statements about experienced or observed symptoms.	*„Well, I my gums bleed. And the gums are receding. And the fear of loosing teeth is looming.“ (Patient ID: 10_53, male, in supportive therapy)*
Definition	How participants define the disease for themselves; their own mental model of the disease.	*„For me, periodontitis is a disease mainly of the gums. But of course I know that it also affects the periodontium. That's the case for me now. It's quite severe. But for me, it's really just a disease of the gums.“ (Patient ID: 03_33, female, in supportive therapy)*
Perception in the public	How participants perceive periodontitis is discussed within the general population.	*„I do not think periodontitis is discussed appropriately in the general population. We have data, from different studies, that show that the epidemiological burden in the population is high.“ (Expert ID: 07, dentistry, non-university research institution)*
Awareness of the severity of the disease	The extent to which participants either understand their own disease or experts' perception of patients' understanding.	*„ And although they know that there are interactions with other diseases, some of them [patients] focus on completely wrong diseases. So, the fact that there are connections to cardiovascular diseases, to diabetes, so to speak, this medical or further medical aspect, periodontal diseases, patients often don't have that, at least not at the beginning. And I think that's very important information, because I believe that once you realize that the disease doesn't just affect the oral cavity, but can somehow affect the whole body, that would significantly increase awareness and understanding of the disease on the part of patients.“ (Expert ID: 07, dentistry, non-university research institution)*<br><br>*„For example, if we talk on the phone today and bring up the subject [of periodontitis], then I know exactly where my problem areas are and where it hurts and causes discomfort. And this fear that the status could change*

		at any moment, and that I could lose more teeth […], is simply always there.“ (Patient ID: 12_50, female, in supportive therapy)
Chronic disease	Discussions about the chronic character of periodontal disease.	*„My periodontal therapy is now completed after the big surgery.“ (Patient ID: 07_111, female, in supportive therapy)*<br><br>*„It is not so easy in periodontology, as it is a chronic disease with lifelong need for professional care.“ (Expert ID: 07, dentistry, non-university research institution)*
Uncertainties	Aspects of the disease that were not well understood	*„Perhaps where exactly periodontitis develops. I haven't looked into that yet. Whether it's more likely to be the tooth root or the nerves. Whether it's really in the gums, I'd say as a layman, the fleshy part, where bacteria might then... I don't know about these things. But I wasn't interested enough to look into it.“ (Patient ID: 05_51, female, in supportive therapy)*
**Self-effectiveness as a patient**	To what extent patients perceive they can influence the outcome of their periodontal therapy	*„But for me as a patient, yes, it's completely sufficient if I go to the dentist now, he takes a look, he does the periodontal treatment if necessary. Then I go home satisfied.“ (Patient ID: 08_119, male, in supportive therapy)*
**Treatment in the dental practice**	Statements on how the treatment in the dental office is perceived; topics related to the therapy itself.	
Fear	Patient attitudes towards self-monitoring with a smartwatch before the study	*„I'll just say it straight out: I don't like going to the dentist. I'm afraid of the dentist, but probably also... I can't say exactly why. I don't like the instruments, I don't like the smell. All the poking around in my gums and around my teeth is very unpleasant and painful for me, even if they numb it a little.“ (Patient ID: 05_51, female, in supportive therapy)*
Qualities of the practitioner	Patient statements on the unique qualities of their treating dentists.	*„The reason is really because our dentist is simply—I always say he's one of the most important men in my life. (Laughter) He has helped me so much and supported me so well that my teeth are now functioning and preserved as they are today.“ (Patient ID: 12_50, female, in supportive therapy)*
Perception of treatment	Statements about how the periodontal therapy is received in general.	*„Treatments have been changed from time to time because of new developments in technology or medicine. Adjustments were made, and if they worked, they were retained. However, there are one or two things*

		that we did not pursue further, but instead reverted to the old way, so to speak.“ (Patient ID: 04_67, female, in active therapy)
Treatment outcome	How the result or outcome of the treatment was perceived.	*„Absolutely, my dentist too, so I can clearly see that my gums no longer bleed when I brush my teeth, that I no longer have the feeling that my gums are so spongy, it was such a strange feeling in my mouth, on my gums, It's hard to describe, but it was a spongy feeling and you had the impression that you couldn't bite properly anymore because everything was kind of receding, and luckily that hasn't been the case for a long, long time now.“ (Patient ID: 07_111, female, in supportive therapy)*
**Caring for patients with periodontitis**	Statements about the organisation of periodontitis care in Germany.	
Lack of evidence	Statements about lack of evidence on periodontitis therapy	*But if we discuss about, for instance, the systemic link, you know, the amount of studies on this and how much blood was taken to evaluate whatever disease with periodontal disease, that’s tons, tons, tons of money, large effort, whatever. […] And then we’re not even taking into account where the money goes when it comes to if you lose your teeth. The amount of studies that have been done on dental implants. And there are not even ten studies on how to maintain implants by self-care. (Expert ID: 17, dentistry, non-university research institution)*
Financial aspects	Statements about financial aspects of receiving or providing periodontal therapy.	*„The only thing, as I said, but that has nothing to do with our dentist. I think that going 3ss et dentist is an expensive undertaking, and so is teeth cleaning or care or whatever. That‘s the only thing, that‘s a big criticism, but I don‘t blame the dentist 3ss et3t. It‘s just that our statutory health system 3ss et up wrong. I mean, I‘d rather pay a little more in my health insurance premiums and just have peace of mind.“ (Patient ID: 05_51, female, in supportive therapy)*
Reimbursement for dental care providers	Reimbursement for provision of periodontal therapy	*„So there are reasons why communication between patient and dentist is not happening well. And one of the reasons is that normally there is no reimbursement models, so the dentist is expected to educate the patient without being compensated for his time.“ (Expert ID: 11, dentistry, academia)*

Cost	Costs associated with receiving periodontal therapy	*„Yes, but when it hurt so much and they [sessions] are so long and the financial outlay, because with a renovation like that, it easily costs 8,000 euros, which you don't always have as a pensioner, so you had to give up your vacation, then you take it very seriously.“ (Patient ID: 16_83, female, in supportive therapy)*
Interprofessional exchange	Statements on exchange, e.g., of information, between different professions.	*„There needs to be a bit more fluidity between the two—dentistry is no longer just dentistry, and medicine is no longer just general medicine. Because we know that with Alzheimer's, diabetes, heart disease, and other conditions, bacteria are involved. And it would be beneficial if patients were referred back to cardiologists for regular check-ups.“ (Expert ID: 06, oral hygiene, private dental practice)*
Role of the dentist	Experts‘ description of dentists‘ role in successful periodontal therapy	*„And I think we have to move our mentality from a surgical type of approach to periodontitis care, periodontal care to a medical one where it is very much about empowering the patient, about educating the patient and getting the building blocks right.“ (Expert ID: 05, dentistry, academia)*
Communicating with patients	The influence of communication style and relationships with patients	*„We were well known for, you know, "you don't brush good enough". You know, "you need to do better", on a very negative way. And I think, we luckily are moving a bit away from that negative point. So, we need to empower them and the things that were good. And just give some tips, you know. "If you put your brush a little bit like this or if you use your interdental brush a little bit more like that. Or did you think about using it from that side or did you consider-? Or maybe this type of interdental brush will enhance, will obtain a better effect." I think this personal positive feedback with tips, this is what I've seen during my career that really helps patients. Not general statements like: "Brush twice a day with a fluoride toothpaste." (Expert ID: 17, dentistry, non-university research institution)*
Effectiveness of the treatment	How treatment success can be sustained	*„It is certainly also necessary to empower patients, during or between professional interventions, in the sense of supportive periodontitis therapy, so that they do their best. I am thinking of interdental spaces, effective interdental cleaning, and various methods of biofilm management at home.“ (Expert ID: 07, dentistry, non-university research institution)*

Patient-specific factors	Factors contributing to treatment success on an individual level	*„Of course, we have limitations that we cannot circumvent when there are skill deficits because my patient is simply no longer as skilled as they used to be or never was skilled in the first place. So when there are manual problems that... Many people are very motivated, but in the end they are still not effective because it doesn't work.“ (Expert ID: 03, dentistry, academia)*
Evaluating treatment success	Factors used to determine treatment success	*„The issue is the gaps between the teeth. It's quite obvious, you can see it with me too, that my teeth have shifted so much that I now have much larger gaps between my teeth, and gaps. Firstly, this is obviously an aesthetic issue. Secondly, however, I am certain that, and this is something you discuss with others, that abroad, for example, the focus is different, that something is done to prevent this. Perhaps with something that also plays into aesthetics, for example. And at the same time avoids me having to hold that little brush in my hand 15 times a day.“ (Patient ID: 12_50, female, in supportive therapy)*
Changes	Perceived changes in the treatment of periodontitis	*„In any case, it seems relatively obvious to me, even though I don't have any hard data to back this up, that younger colleagues are already focusing more on periodontology in general.“ (Expert ID: 07, dentistry, non-university research institution)*
Shortages	Perceived shortages in access to or provision of periodontal care	*„At least, if you look at the claims data, periodontal screening is not performed and billed often enough.“ (Expert ID: 08, dentistry, academia)*
Reasons for shortages	Reasons for shortages in access to or provision of periodontal care	*„The political level is yet another aspect and here the major aspect is that public health in dentistry has been historically dominated by people interested in caries. And the focus has always been on the prevention of caries.“ (Expert ID: 11, dentistry, academia)*
**Switching/Changing dentists**	Experiences with changing the treating dentist in the past	*"Well, I found it alarming that my dentist didn't recognize this and dismissed it with, "Yes, it'll be fine. Brush more, do more, it'll be fine." But he didn't do anything about it. So, I found that quite alarming. But thankfully, I found someone else and switched. And I'm really glad I did, really glad. So, yes, that's really, really important." (Patient ID: 18_51, female, in supportive therapy)*

**Need for information**	Statements referring to information needs or previous experiences with researching information on periodontitis	*"And quite often, the patients themselves, if they're really motivated, their level of understanding increases dramatically during treatment. So the questions they'll ask at the beginning of treatment are very basic questions, such as how long do I have to brush for, how many times a day, does it matter what type of toothpaste I use, does it matter what type of toothbrush I use. Those types of questions. Once they've been through treatment, you know, the really motivated patient will want to know about their upper left second molar because they remember there were some deep pockets there and have those deep pockets now reduced to a shallower size. They develop that level of insight and understanding. And I think you know you've achieved something when you get that level of understanding." (Expert ID: 05, dentistry, academia)*
Strategies for information retrieval	Concrete strategies of patients to research information on periodontitis	*"And, yes, as I said, it just got worse and worse. And I thought, something's not right here, there must be something wrong. And then you start googling a bit to see what it could be, what's causing it" (Patient ID: 18_51, female, in supportive therapy)*
Information provided by the dentist	Patients' recollections on how they received information from their dentist	*"I did a little reading, including some flyers that my dentist gave me." (Patient ID: 13_58, female, in supportive therapy)*
**Oral hygiene routines**	Statements on performed oral hygiene (routines)	
Tongue cleaning	Reports about cleaning of the tongue	*"From time to time, but less regularly, I use a tongue cleaner." (Patient ID: 14_101, male, in supportive therapy)*
Difficulties	Difficulties experienced when performing oral hygiene	*"And dental floss, but not everywhere, because I have fillings where I can't really get through, and I'm afraid that a little bit will break off." (Patient ID: 13_58, female, in supportive therapy)*
Mouthwash	Reports about using mouth rinse	*"I always use mouthwash when I brush my teeth, and sometimes in between. I always have a small bottle in my handbag. I got into the habit of doing this because I used to have really bad bad breath. Yes, and every now and then I use this new gel and mouthwash when I feel like I might be getting an infection again or something might be coming on." (Patient ID: 13_58, female, in supportive therapy)*

Oral hygiene instructions	Statements about receiving or giving instructions for performing oral hygiene	*"The second thing, I believe, is to simply explain oral hygiene to patients. In other words, the use of aids. And this must be explained in the dental practice, either by a professional prophylaxis specialist or by the dentist. Otherwise, if you do it yourself at home without guidance, you can cause more damage." (Expert ID: 10, dentistry, professional association)*<br><br>*"He [the dentist] told me exactly what I had to do there, how I had to maintain it every day. And I stick to that very strictly." (Patient ID: 18_51, female, in supportive therapy)*
Motivation	Statements about motivation to perform oral hygiene	*"And so my awareness has slowly expanded, and even beyond the dental restoration, which turned out really well, and beautiful bright teeth, which is simply part of it now, it's important to take good care of them. And I never want to lose that again. So I want to at least maintain the standard I've now set for myself and keep all my teeth. And this awareness, it just didn't exist before." (Patient ID: 23_31, female, in supportive therapy)*
Burden	Statements about the perceived burden of performing oral hygiene	*"It is burdensome to use interdental brushes to perform such an intense cleaning." (Patient ID: 03_33, female, in supportive therapy)*
Barriers	Barriers to performing oral hygiene	*"The social environment definitely has an influence. Whether in the family, whether I'm the only one standing in the bathroom for half an hour picking at my teeth..." (Expert ID: 08, dentistry, academia)*<br><br>*"Yes, I think perhaps financial support as well, because, as I said, it is quite an expensive undertaking. The mouthwash, the sticks, the floss—it all costs money. Of course, I change them regularly. I can't use these sticks for, say, three days in a row. I change them every two days at the latest and then use fresh ones. Yes, it does cost money; a packet costs four or five euros. Of course, I have different sizes because my gaps are also different sizes. So, I'd say you have to reckon with around 15 to 20 euros for these sticks each time. And this solution, yes, it's not exactly cheap either. It all adds up. And I think that for many people, this is perhaps the reason why they don't do it*

		regularly, because they simply don't want to sacrifice that much." (Patient ID: 18_51, female in supportive therapy)
Satisfaction with routines	Statements about satisfaction with current oral hygiene routine	*"I would say that the teeth are fine, so the routines seem to be effective or sufficient." (Patient ID: 22_57, male, in supportive therapy)*
Change of routines	Statement about changes in oral hygiene routines	*"I did that more quickly, whereas now, after this periodontitis treatment, I do it very meticulously and take my time. So, you learn from your mistakes." (Patient ID: 16_83, female, in supportive therapy)*
Interdental cleaning	Statements about performing interdental cleaning	*"And I always carry an interdental brush and dental floss with me so that I can clean between my teeth immediately after eating." (Patient ID: 12_50, female, in supportive therapy)*
Brushing	Statements about brushing teeth	*"So I brush my teeth at least twice a day. For at least two minutes. I use a toothbrush with a round head. And then I really try to brush every segment of my jaw area for half a minute, without applying too much pressure." (Patient ID: 10_53, male, in supportive therapy)*
**Prevention**	Discussions about the prevention of periodontal disease	*"So all this individual prophylaxis that children are taught focuses solely on caries. There is a passing reference somewhere that you should also start cleaning between your teeth at some point, teaching children how to clean between their teeth, but I don't think there is a concept for this." (Expert ID: 07, dentistry, non-university research organization)*<br><br>*"If I had known ten years ago that I had such pockets, and that this was due to poor dental hygiene, which does not refer to brushing my teeth, but to cleaning them with other aids. If I had known that, I would have been spared a lot of trouble." (Patient ID: 03_33, female, in supportive therapy)*
**Digital tools in dentistry**	Statement about different aspects of digital tools in dentistry in general	
Previous experiences with digital tools in dentistry	Previous experiences with digital tools such as mHealth	*"So we also have the toothbrush. And then there's the app, which shows which areas you've brushed, that you've really covered every area, and how long you've been brushing. Although two minutes is never enough for me, so I brush for at least three." (Patient ID: 13_58, female, in supportive therapy)*

Barriers	Barriers to using/implementing digital tools in dentistry	*"But almost every time when people use digital technology or when policymakers or companies or something like that want to use digital technology, they increase the inequality to access to care. It means that just people who are able to buy a smartphone or to pay for an app or to pay to use an app, that could be free at the beginning, will be able to use this system." (Expert ID: 01, dentistry, academia)*
Scope	Tasks/groups which could be supported by the use of digital tools	*"But initial results show that vulnerable groups as a whole simply benefit from this app. This includes people who do not have a migrant background but have a low socioeconomic status. They benefit equally from this digital opportunity." (Expert ID: 09, dentistry, academia)*
Benefits	Potential benefits of using digital tools	*"If you want to learn [oral hygiene], you need to learn in a safe environment what is home for patients." (Expert ID: 17, dentistry, academia)*
**Digital Health Companion**	Statements about a patient-facing digital health companion	
Fun/motivation	Implementing fun and motivational aspects in the app	*"Make it fun. Because brushing, oral hygiene is not fun. So, if you can turn this into a fun and enjoyable activity you are going to have people more motivated. Not just children, adults too." (Expert ID: 15, mHealth, industry)*
Challenges	Suggestions to implement a challenge in the app	*"Yes, you could do that through competitions, right? You could also organize a competition in your practice. The cleanest tooth, right?" (Expert ID: 12, dentistry, academia)*
Setting goals	Suggestions to allow patients to set goals for themselves in the app	*"And maybe say, okay, I'll make a little agreement with myself that I want to use it every day, and then I'll experience success […]. So, okay, I have certain intermediate goals that I want to achieve somehow, which don't have any health outcomes at first, but which contribute to using the app." (Expert ID: 16, mHealth, academia)*
Reward system	Suggestions to implement a reward system in the app	*"And then I have to get some kind of goodie. That is, a little reward. Something along the lines of: "The more accurately you enter the data, the more accurately we can help you" or something like that." (Patient ID: 10_53, male, in supportive therapy)*

Gamification	Suggestions to implement little "games" in the app	*"Sure, gamification, if you mean that you can collect points for good behavior, is always a good thing in principle. That's what we call token economy. And you don't even need any financial incentives or anything like that. Just the fact that I've noticed that I've somehow finished a flower in my thing has a self-reinforcing effect." (Expert ID: 13, dentistry, non-university research institution)*
Usage in general	Different aspects about using an app to support periodontal therapy in general	
Ad-free	Statements about ads in the app	*"Sometimes, you're watching something and then at the end there's always some story telling you to buy something or whatever. I think that's really annoying. That's why I don't even bother watching it." (Patient ID: 20_64, female, in supportive therapy)*
Barriers	Potential barriers to using the app	*"That if I already have the app on my phone, it's just open for me. That I can access it immediately." (Patient ID: 05_51, female, in supportive therapy)*
Suitability for daily use	Factors complicating potential everyday use	*"So we haven't done that in general but what we do know personally from our app and our technology is that one of the barriers is bringing the app through the phone into the bathroom during the brushing. So that like creates an extra step." (Expert ID: 15, mHealth, industry)*
Complex log in	Statements relating to log in procedures	*"Yes, I find that annoying. I don't find it particularly encouraging to use an app when every time I have to enter user data or, which you can't remember anyway and have to look up, it makes the app unattractive to me." (Patient ID: 05_51, female in supportive therapy)*
Discoverability	How patients could have difficulties finding the app or being informed about its existence	*"So if you say, "I have a website where you can download it," that's not likely to happen. You have to embed it in the consultation and support process. It can also involve a great deal of explanation." (Expert ID: 16, mHealth, academia)*<br><br>*"Well, I can't really imagine that at the moment. If something like that existed, yes, someone would have to tell me about it and show me what I*

		should or could do. Personally, I don't know much about special apps like that." (Patient ID: 08_119, male, in supportive therapy)
Age	Age as a potential barrier to app use	*"That's why I would personally reject the app for myself, but I wouldn't reject it for younger people in general. I would say that it's also an option that you have. But I'm not very tech-savvy." (Patient ID: 16_83, female, in supportive therapy)*
Cost	Cost as a potential barrier to app use	*"As long as it is free and the app costs nothing or very little." (Patient ID: 18_51, female, in supportive therapy)*
Data privacy	Data privacy as a potential barrier to app use	*"It would probably be very important for such an app to provide information about data protection and so on, and to be secure in some way. Because you're sharing relatively sensitive data. I have that in my own professional environment too. So you have to be very sensitive in some way, and I think at least some of the potential users would be too." (Patient ID: 14_101, male, in supportive therapy)*
Intent to use	Statements about the intent or interest in using an app to support periodontal therapy	*"So what is the motivation for the patient to use the app? So there needs to be something driving them to you." (Expert ID: 15, mHealth, industry)*
Existing intent to use	Positive statements about an existing intent or interest to use the app	*"I don't think that's a bad idea. There are apps for everything imaginable, apps for everything unimaginable, and this would actually be something really useful. I would support that, I think it's a good idea." (Patient ID: 23_31, female, in supportive therapy)*
No intent to use	Negative statements about intent or interest to use the app	*"So for me, that would certainly not be particularly helpful or necessary. No, I would not use it." (Patient ID: 03_33, female, in supportive therapy)*
Technical aspects	Statements about the technical aspects	
Offline usage	If the app can be used without an internet connection or not	*"So the question is, what are the general conditions? Can it also be used offline? Do I always need the internet?" (Expert ID: 16, mHealth, academia)*
Connection to electric toothbrushes	Interoperability with electric toothbrushes	*"Or, let's say, completely digital, or connected to a device in the bathroom. In other words, my electric toothbrush, for example, if it could do something like that." (Patient ID: 14_101, male, in supportive therapy)*
Chatbot	Availability of a chatbot	*"The ideal situation would be, of course, to enter a question and then get an answer. Or I could search for questions and then get answers. […] When*

		you're researching on the internet, you always have to read a lot. And it would be helpful if you could somehow retrieve the information in a more targeted way. For example, I could enter a keyword and then get information instead of having to read through everything first to find the answer to my question somewhere." (Patient ID: 21_58, female, in supportive therapy)
Handling	Aspects regarding the handling of the app	*"Of course, it must not be used in such a way that I then have to spend an immense amount of time filling the app." (Patient ID: 21_58, female, in supportive therapy)*
Customizability	Opportunities to customize the app	*"I would be more likely to respond to video material. But maybe I would also switch to text if I wanted to. So offering options for how I want the information to be presented. Personally, I would prefer a video. Short clips. Not long, just short clips." (Expert ID: 08, dentistry, academia)*
Language	Use of appropriate language	*"Then, of course, there is the question of complexity in language. Are there perhaps different levels when it comes to language?" (Expert ID: 16, mHealth, academia)*
Accessibility	Options to improve accessibility	*"Especially if they are older patients, the question of accessibility always arises. They usually have certain visual impairments. And does it still work well when I look at it with 200 percent magnification?" (Expert ID: 16, mHealth, academia)*
Push notifications	Statements regarding the implementation of push notifications	*"Depending on what this app is supposed to do, but with reminder functions, these push notifications, we were a little concerned that patients would find them rather negative. Now another message, but in fact it was received rather positively. So just a reminder about the dentist appointment, remember, you have to make an appointment in three weeks. That was rather an advantage." (Expert ID: 09, dentistry, academia)*
Suggestions for app functionalities	Statements about potential app functionalities	
Reminders for appointments	Option to receive a reminder for an appointment	*"But I think this app, which reminds you via app or text message, is a good way to go, because you might forget something once in a while, and if it reminds you the day before, then you know for sure that you have the*

		appointment the next day." (Patient ID: 05_51, female, in supportive therapy)
Making appointments	Option to make appointments from the app	*"Yes, I could imagine that, if it works in conjunction with the dental practice, in conjunction with the dentist, because he is the one who makes the appointments." (Patient ID: 15_44, female, in supportive therapy)*
Analysis of user data	Learning from documented user data	*"So I see the advantage when something is evaluated in the background by (you), i.e. by the app operators, and they say: "Okay, so we've found that cleaning between your teeth three times a day is better than twice a day." Then you say: "Okay. Maybe I'll take that as a starting point." Or that it's better to use a thinner brush or a thicker interdental brush. So, advice like that, where you've basically gained some experience from the million app users, which many people have had independently of each other. And then you make that available to everyone else." (Patient ID: 10_53, male, in supportive therapy)*
Daily oral hygiene	Suggestions to support daily oral hygiene routines	
Brushing timer	Implementing a timer to track brushing time	*"The only thing I might need is a timer so that I can use the time for dental hygiene, brushing, and so on, accordingly." (Patient ID: 03_33, female, in supportive therapy)*
Diary	Implementing a diary to document performed oral hygiene	*"I don't know if I would do it now, because I don't feel the need to, but if I wasn't feeling so good right now or didn't have my routines, I would keep a kind of self-observation diary, in other words, simply record things like, to put it bluntly, did my gums bleed or not, or something like that. So, a kind of diary or whatever, to perhaps simply document any complaints until the next treatment or something. I would find it practical, I would use it in an emergency, if necessary." (Patient ID: 14_101, male in supportive therapy)*
Reminders	Implementing a reminder to remind about oral hygiene	*"If you have an app that reminds you every day have you brushed your teeth, have you flossed, have you done this. I mean all the right things. That could probably work." (Expert ID: 04, dentistry, academia)*

		“That you have that as support, as support for your own schedule, so to speak. That it might also say, “Have you gotten your new toothbrush yet?” Or “Have you changed your toothbrush?” or things like that. Things that you should do, things that everyone knows you should do, like changing your toothbrush after a certain amount of time. But when is that time? And then you think: “Okay, I last changed it six months ago. So now it's time again.”” (Patient ID: 10_53, male, in supportive therapy)
Smoking cessation	Implementing the topic of smoking cessation in the app	*“I think it should be tailored to me individually. As I said before, if I were a smoking patient, I should be offered this [information on smoking cessation] or that in some way.” (Expert ID: 08, dentistry, academia)*
Augmented reality	Implementing augmented reality in the app	*[in photos] “Yes, or mark the things that have improved with color coding. So that you can see it at first glance.” (Patient ID: 06_56, male, in active therapy)*
Connection with other patients	Statements about possible connections to other perio patients through the app	*“Self-help groups aren't for everyone, but if you can interact with a community, that's always a good thing. You can definitely consider that as well.” (Expert ID: 13, dentistry, non-university research institution)*<br><br>*“No, I've read enough comments on medical topics (?), it's horrible what people say to each other. For God's sake, no.” (Patient ID: 06_56, male, in active therapy)*
Summary of last dentist visit	Statements about the potential of a summary for each dentist appointment	*“And that's why I think it would be good if, after these professional impulses, the app could help you summarize them and say, “OK, so now it would make sense for you to do this and that.” (Expert ID: 07, dentistry, non-university research institution)*
Tailoring	Ideas for potential ways to tailor app contents to individual user needs	*“I think personalizing the message is the most important thing. I think making the patient feel as an individual, that everything you're telling them is about them and is specific to them is really important. And that's where I think digital technology can come in. I think, you know, presenting patients with their risk scores, presenting them with the scale of the amount of disease they have, maybe using a traffic-like system so they understand that green is good, and red is perhaps not so good. That type of*

		information can be really important, almost like a scale or a barometer of their periodontal health, showing gradually that it's improving with time, I think that's motivational to patients." (Expert ID: 05, dentistry, academia)<br><br>*„And since routines have been agreed upon, which I may have discussed with my dentist, as I said, first the one-pager, this is what my teeth look like, this is green, that's great, this is yellow, that's red, you need to work on that." (Patient ID: 22_57, male, in supportive therapy)*
Progress visualization	Importance of visualizing progress	*"But also I guess having the option of you know as they build their confidence and motivation they should be able to see how they improve and that will hopefully provide additional motivation." (Expert ID: 15, mHealth, industry)*
Presentation of successes	Methods of presenting successes	*"So what we haven't talked about yet is coloring something and showing how well I've cleaned. Feedback on improvements in condition and highlighting problem areas. So, if that accompanies me through therapy and I have problem areas where I need to brush better or pay special attention, then it might also be helpful for patients if you can show them that again in a picture, for example. Showing that and, of course, displaying successes in the app as well." (Expert ID: 13, dentistry, non-university research institution)*
Clinical measures	Statements on the inclusion and presentation of periodontal outcome parameters	*"Some people think it's really cool that you have to link the findings to this. There are patients who always want to take their probing status home with them so that they can see what has improved and what has worsened, and can check again. But there are very few of them." (Expert ID: 03, dentistry, academia)*
Photographic documentation	Statements about the possibility to document photos	*„I really think it's very good. Because, I mean, my dentist didn't take any pictures. But I noticed every time that it really looked much, much better, the gums themselves. There was real progress after every treatment. That was really great. And I think it's good that you can see before, after, or before and the current state now, or something like that, I think that's really good, yes." (Patient ID: 18_51, female in supportive therapy)*

Direct interaction with treating dentist	Statements about possibilities for direct interaction with the treating dentist through the app	
teledentistry	Opportunities for consulting patients at their home	*“Where I can make contact with the patient, right? Or even, we're not there yet, but that will come eventually, right? That you can make certain findings via the camera, via a sensor, or via something else. Those could be ways.” (Expert ID: 12, dentistry, academia)*
chat	Option to chat with the provider to ask questions	*“Emergency contact for the dentist. Help, my tooth has broken out after all. The splint didn't work, yes. I need a new everStick. Something like that, maybe get in touch there too.” (Expert ID: 08, dentistry, academia)*<br><br>*“If you ask a question now, it's probably unrealistic to think that you'll get an answer within an hour. But it should be possible within a day. So if you already have an app, you can maintain it and have someone who can answer questions like this.” (Patient ID: 15_44, female, in supportive therapy)*
Information about periodontal disease	Statements on what kinds of information should be included and how the information should be presented	
Reliable information	The app as a source for reliable information	*“Because you know that the source is reliable. Not everything on the internet is always accurate. So you have to be careful about which websites you read and whether you accept the information or not, and I think that if it's in an app that has been specially designed by experts, then the data sources are reliable.” (Patient ID: 13_58, female, in supportive therapy)*
Presentation of information	Different ways for attractive presentation of information about periodontitis	
Lightbulb moments	Creating lightbulb moments for patients	*“Otherwise, these apps offer something that is completely natural for us, but may be a revelation for patients. So it's not just about communicating risks, which could be highlighted from time to time, but also how a*

			successfully treated periodontitis can improve quality of life." (Expert ID: 03, dentistry, academia)
	Information snippets	Information presented in small sections	*"Just throwing in random facts like that. As simple and visually appealing as possible, because that way you can take them in without any effort. So, the short period of time that exists in which people continue to scroll and swipe, so that the information can be grasped within a maximum of five seconds. That would be totally cool." (Expert ID: 03, dentistry, academia)*
	Comprehensibility	How information has to be presented to be comprehensible	*"Yes, I think it shouldn't be too medical. It should be written in a way that everyone can understand. It should be a little simpler, easier to read. Because when it gets as difficult as it just did, people click away." (Patient ID: 20_64, female, in supportive therapy)*
	Visualization	How information should be visualized	*"So just text, I wouldn't want that. It would be nice with pictures or maybe even short videos." (Patient ID: 09_54, female, in supportive therapy)*
Need for information		Topics that should be addressed; Gaps in knowledge	*"An effective app should be able to educate the patient." (Expert ID: 15, mHealth, industry)*
	Therapy journey	Information about the current position along the therapy journey	*"I think that makes sense when patients know what stage of therapy they are currently at. Periodontitis treatment is very long. And that way they can follow their own progress a little bit." (Expert ID: 03, dentistry, academia)*
	Specialized dentists	Information about specialized dentists and how to find them	*"I think that's very important, very important. Because, ultimately, as a patient, you have no idea. So, as a rule, most people have nothing to do with medicine. You trust the person, you're there, you get help if you have a hole in your tooth or something else is wrong. Yes, I think a checklist like that is very, very important." (Patient ID: 18_51, female, in supportive therapy)*
	Oral hygiene instructions	Instructions about performing oral hygiene	*"But illustrated texts are fine. In short, I think that's okay. If the toothbrush is supposed to be held at an angle, then there should be a little picture there so I can see what is meant. Or with these interdental brushes, I have them angled upwards and not downwards, because otherwise I'll damage the gums between the teeth. But, as I said, I think illustrated pictures would be sufficient, videos would be a bit boring. For me personally, anyway." (Patient ID: 10_53, male, in supportive therapy)*

	Product overview	Overview of different available tools for oral hygiene	*“I think it would be good if the app also showed all the different types of interdental brushes available. Because I think these brushes are really effective in treatment. And if you don't google or search or look, then you don't know what's available. Like what kinds of brushes there are, what handles you can get. Yeah, you could see it as advertising. So, we shouldn't overdo it with advertising brushes, but simply give those who brush their teeth the opportunity to see that there are other options besides those available at drugstores or similar stores. Because I didn't even know about the brushes I now have for my periodontal treatment until recently.” (Patient ID: 13_58, female in supportive therapy)*
	Myths	Provide clarification about existing myths regarding periodontitis	*“Myths. Well, it just occurred to me that maybe you could turn something like this into a game, like a quiz? A quiz, “what do you think,” and then somehow verify or question myths that way. Yes. Even I would actually enjoy that.” (Expert ID: 06, dentistry, private practice)*
	Causes	Need for clarification regarding causes for developing periodontal disease	*“So, they take a lot of personal responsibility, they believe it's their fault, they don't understand the genetic basis and they also don't understand why they're different from other people when it comes to levels of plaque control, they need to achieve.” (Expert ID: 05, dentistry, academia)*
	Research updates	Provide up-to-date information from latest research	*“Exactly. What has happened in research now, what has happened at all by looking at it this time, what has happened there. And if you then realize, “Okay, this interdental brush doesn't do anything. We're going back to dental floss,” because it's much better, that would also be a result.” (Patient ID: 10_53, male in supportive therapy)*
	Explaining symptoms	Need for explanation of periodontitis symptoms	*“I think you have to combine it with visual material so that people can recognize themselves. So that they understand what it means when the gums detach from the teeth and these pockets form, what the bacteria do in there, so that you can visualize it a little, not just text. But also with pictures and simple language so that laypeople who aren't so deeply involved can understand it better.” (Patient ID: 13_58, female in supportive therapy)*

	After appointments	Tips on how to deal with pain/discomfort after therapy	*"Care tips and then maybe just that after the first treatments, that they are actually allowed to eat and stuff like that, like saltwater rinses." (Expert ID: 06, dentistry, private practice)*
	Associated diseases	Information about diseases associated with periodontitis	*"I just think it's important to point this out to patients with periodontal disease: Hey, go see your general practitioner to have your blood sugar levels checked, because you have a threefold increased risk of developing diabetes. […]. To what extent this should be expanded in the app, I wouldn't go too far. But I do think it's important to point out that periodontal disease affects more than just the mouth." (Expert ID: 10, dentistry, professional association)*
	Nutrition	Information about nutrition during periodontal therapy	*"This tooth-healthy diet is something that the app should also try to reflect. Because, as we all know, diet and periodontitis play an important role. I think that would be a good thing." (Expert ID: 09, dentistry, academia)*
